# Pathways into mental health care for UK veterans: a qualitative study

**DOI:** 10.1080/20008198.2017.1389207

**Published:** 2017-10-25

**Authors:** Harriet Mellotte, Dominic Murphy, Laura Rafferty, Neil Greenberg

**Affiliations:** ^a^ Doctorate in Clinical Psychology, Addiction Sciences Building, Institute of Psychiatry, Psychology and Neuroscience, King’s College London, UK; ^b^ King’s Centre for Military Health Research, Weston Education Centre, King’s College London, London, UK; ^c^ Combat Stress, Tyrwhitt House, Leatherhead, Surrey, UK

**Keywords:** Veterans, ex-service personnel, mental health, stigma, barriers, help-seeking, Veteranos, personal que ya no está de servicio, salud mental, estigma, barreras, búsqueda de ayuda, 老兵, 退役人员, 心理健康, 污名效应, 障碍, 寻求帮助, • This article aimed to understand why a vast majority of veterans suffering from mental health difficulties do not seek professional help. • Living with untreated mental health difficulties has significant negative implications for individuals, society and the economy. • During interviews with veterans suffering from mental health difficulties, we learned about a number of barriers which got in the way of them accessing help, and a number of enablers which allowed them to get the help that they required.

## Abstract

**Background**: It is well established that veterans suffering from mental health difficulties under use mental health services.

**Objective**: This study aimed to understand more about the barriers that prevent veterans from seeking professional help and the enablers that assist veterans in seeking professional help. It also aimed to explore potential mechanisms to improve veterans’ help-seeking and pathways to care.

**Method**: The study employed a qualitative design whereby 17 veterans who had recently attended specialist veteran mental health services took part in semi-structured interviews. The resultant data were analysed using grounded theory.

**Results**: Participants described two distinct stages to their help-seeking: initial help-seeking and pathways through treatment. Specific barriers and enablers to help-seeking were identified at each stage. Initial barriers included recognizing that there is a problem, self-stigma and anticipated public stigma. Initial enablers included being in crisis, social support, motivation and the media. Treatment pathway barriers included practical factors and negative beliefs about health services and professionals. Treatment pathway enablers included having a diagnosis, being seen in a veteran-specific service and establishing a good therapeutic relationship. Participants provided some suggestions for interventions to improve veterans’ help-seeking in future; these focussed on enhancing both veterans and health professionals’ knowledge regarding mental health difficulties.

**Conclusions**: This study identified a number of barriers and enablers that may impact a veteran’s journey in seeking help from professional services for mental health difficulties. Enablers such as reaching a crisis point, social support, the media, having a diagnosis of PTSD and veteran-specific mental health services appeared to be important in opposing stigma-related beliefs and in supporting veterans to engage in help-seeking behaviours.

## Abstract (es)

**Planteamiento**: Está bien establecido que los veteranos que sufren de problemas de salud mental infrautilizan los servicios de salud mental.

**Objetivo**: Este estudio estaba dirigido a comprender más sobre las barreras que impiden que los veteranos busquen ayuda profesional y aquello que les facilita su búsqueda de ayuda profesional. También tenía como objetivo explorar posibles mecanismos para mejorar la búsqueda de ayuda y las vías que llevan a dicha atención de los veteranos.

**Método**: El estudio empleó un diseño cualitativo mediante el cual 17 veteranos, que habían asistido recientemente a servicios especializados de salud mental para veteranos, participaron en entrevistas semiestructuradas. Los datos resultantes se analizaron utilizando una teoría fundamentada.

**Resultados**: Los participantes describieron dos etapas distintas en su búsqueda de ayuda: búsqueda inicial de ayuda; y vías del tratamiento. En cada etapa se identificaron las barreras específicas y los facilitadores para dicha búsqueda de ayuda. Las barreras iniciales incluyeron el reconocimiento de que existe un problema, el autoestigma y el estigma público anticipado. Los facilitadores iniciales incluyeron estar en crisis, apoyo social, motivación y los medios de comunicación. Las barreras a recibir tratamiento incluían factores prácticos y creencias negativas sobre los servicios de salud y los profesionales. Lo que facilitaba el recibir tratamiento incluía tener un diagnóstico, ser vistos en un servicio específico para veteranos y establecer una buena relación terapéutica. Los participantes proporcionaron algunas sugerencias de intervenciones para mejorar la búsqueda de ayuda de los veteranos en el futuro; estos se enfocaron en mejorar el conocimiento sobre las dificultades de salud mental. tanto de los veteranos como el de los profesionales de la salud.

**Conclusiones**: Este estudio identificó una serie de barreras y facilitadores que pueden influir en que los veteranos busquen ayuda de servicios profesionales para las dificultades de salud mental. Facilitadores como llegar a un punto de crisis, el apoyo social, los medios de comunicación, tener un diagnóstico de TEPT y servicios de salud mental específicos para veteranos parecían ser importantes a la hora de enfrentarse a creencias estigmatizadas y de apoyar a los veteranos a implicarse en conductas de búsqueda de ayuda.

## Abstract (zh)

**背景：**承受着心理健康障碍的老兵对心理健康服务使用不足，这是普遍存在的。

**结论：**目标：本研究为了更好的理解阻碍老兵寻求更多专业帮助的原因，和能够帮助他们寻求专业帮助的因素。同时为了去探索促进老兵寻求帮助和获取关怀的潜在机制。

**方法：**本研究使用了质性研究方法，17名最近使用过老兵心理健康服务的老兵参加了半结构化访谈。根据扎根理论进行数据分析。

**结果：**参与者描述了两种截然不同的阶段：初始寻求帮助阶段，治疗进程阶段。我们发现了在不同的求助阶段的特定的障碍和促进因素。起始阶段的障碍包括：对出现问题的认知，自我污名效应和预想的公共污名效应。起始阶段的帮助因素有：处在危机中，社会支持，动机和媒体。治疗进程的障碍有：现实因素和对健康机构和专家的负面认识。治疗进程中的帮助因素有：获得诊断，在老兵服务机构治疗，建立良好的治疗关系。参与者提供了一些关于未来能够提高老兵寻求帮助的干预建议，这些建议聚焦于同时提高老兵和健康专家关于心理健康障碍的知识。

**结论：**这篇研究发现了一些影响老兵寻求专业心理健康服务的帮助的障碍和促进因素。帮助因素包括达到危机点，社会支持，媒体，被诊断为 PTSD，专门针对老兵的心理健康机构在对抗污名化信念的过程中起作用，并且支持老兵持续的寻求帮助。

There are around 2.8 million veterans living in the UK (The Royal British Legion, 2014) of which a significant minority experience mental health difficulties (Iversen et al., 2011). In the UK the National Health Service (NHS) is responsible for providing health care to veterans supported by a number of charitable and for-profit organizations. Research suggests that treatment offered by these services is likely to benefit a significant number of veterans were they to access the help (Kitchiner, Roberts, Wilcox, & Bisson, 2012). However, evidence suggests that UK veterans often under use these services (Greenberg, 2014), with only around 50% of veterans seeking help for mental health problems (Iversen et al., 2005). In addition, research indicates that veterans may experience mental health difficulties for as long as 12 years after leaving the Armed Forces (AF) before they seek professional help (Murphy, 2016). Untreated mental health difficulties can be detrimental to veterans’ mental and physical health (Kessler, 2000; Schnurr & Green, 2004), quality of life (Pittman, Goldsmith, Lemmer, Kilmer, & Baker, 2012) and relationships (Shalev, 1997).

Therefore, a number of studies have sought to understand the barriers that prevent veterans from seeking help for mental health difficulties. Findings suggest that factors including stigma (Iversen et al., 2011), negative attitudes about mental health services (Kim, Britt, Klocko, Riviere, & Adler, 2011) and poor recognition of the need for treatment (Britt, Wright, & Moore, 2012) are barriers to help-seeking for veterans. Furthermore, practical barriers to accessing care are deemed important, including problems with the availability and accessibility of services (Brown, Creel, Engel, Herrell, & Hoge, 2011), waiting times (Damron-Rodriguez et al., 2004) and excessive paperwork (Westermeyer, Canive, Thuras, Chesness, & Thompson, 2002).

Thus far research has largely focussed on the role of stigma in help-seeking. Corrigan developed a model in which he distinguished between public stigma and self-stigma (Corrigan, 2005). Public stigma represents the prejudice directed at a group by a larger population. Indeed, research has found that service personnel are more negatively viewed than their peers by military commanders for accessing mental health services (Porter & Johnson, 1994). Self-stigma occurs when people internalize these public attitudes and as a result develop negative beliefs about themselves. Self-stigma is common in military personnel who report not seeking help because doing so would cause them to feel weak (Sharpe, Fear, Rona, Wessely & Greenberg, 2015).

Despite extensive research into the role of stigma in help-seeking, recent quantitative findings have questioned the extent to which such beliefs prevent help-seeking (Sharpe et al., 2015; Vogt, 2011). A systematic review and synthesis of qualitative literature on this topic concluded that there is substantial evidence of a negative relationship between stigma and help-seeking for mental health difficulties within the AF (Coleman, Stevelink, Hatch, Denny, & Greenberg, 2017). Sharpe et al. (2015) hypothesized that in some situations enablers to help-seeking, such as severity of mental health difficulties, may over-ride any stigma-related barriers to care. Research suggests that key enablers to help-seeking for veterans are family and friends (Sayer et al., 2009; Warner, Appenzeller, Mullen, Warner, & Grieger, 2008), having the ability to recognize that one’s symptoms are of psychological origin (Pilkington, Msetfi, & Watson, 2011) and severity of symptoms and level of distress (Kim et al., 2011). In still-serving military personnel, reaching a crisis point was a key enabler to help-seeking (Murphy, Hunt, Luzon, & Greenberg, 2014).

In summary, it is well documented that a number of barriers exist which prevent veterans from seeking help. However, thus far most research has focussed on exploring stigma-related barriers to care in still-serving military personnel and veterans who are not at the time of the research seeking help. In addition, research into the enablers of help-seeking remains scarce. Therefore, the current study aimed to understand the subjective experiences of veterans currently accessing treatment for mental health difficulties, using a grounded theory methodology, in order to understand and generate theory related to barriers and enablers to help-seeking for mental health difficulties in veterans. A secondary aim was to explore, from the veteran’s perspective, what might be done to improve veterans’ help-seeking and pathways to care.

## 1. Method

### 1.1. Design

This study utilized a mixed methods design. Quantitative data regarding participant’s demographic information and current mental health was sought in order to situate the sample and to provide information on their psychological distress. A qualitative design was used to explore veteran’s experiences of help-seeking. Grounded theory was identified as the most appropriate methodology as it allows the researchers to understand the meaning of veterans’ behaviour from their personal experiences in addition to generating new theory based on concepts that emerge from the data (Blumer, 1969). A lack of existing theories to explain veteran help-seeking behaviours meant that using a methodology where new theory can be generated appeared most relevant (Glaser & Strauss, 1967). A purposive sampling strategy was used in this study and, in accordance with grounded theory, the final sample size was dictated once theoretical saturation had been reached and therefore no new or relevant data was emerging and the research questions had been answered (Corbin & Strauss, 2008).

### 1.2. Study setting

Participants were recruited from two specialist mental health services for veterans located in the South East of England: the London Veterans Service (LVS) and Combat Stress (CS). LVS is an NHS veteran-specific outpatient service for veterans living in London; CS is a UK national charity, part funded by the NHS, that provides specialist inpatient and outpatient mental health treatment to veterans. Participants recruited to this study accessing treatment from CS received intensive therapy on an inpatient basis during a three to six week stay. Ethical approval for this study was obtained from the NHS in January 2016.

### 1.3. Participants

Inclusion criteria included: being a veteran (an individual who has performed paid military service for at least one day); having a mental health condition; currently accessing treatment from LVS or CS; and an ability to speak fluent English. Individuals were not included if they had a primary diagnosis of psychosis or a single diagnosis of alcohol dependency as it was not feasible to assess for capacity to consent to participate in the study, or if a health professional deemed their participation in the study clinically inappropriate.

### 1.4. Materials

#### 1.4.1. Standardized measures

Participants completed four brief standardized measures that they returned to the researcher prior to their semi-structured interview. Some participants at CS had already completed the measures as part of routine practice, in which case scores were obtained from the team psychologist to save participant time and effort.

Depression symptoms were assessed with the Patient Health Questionnaire (PHQ-9; Kroenke, Spitzer, & Williams, 2001) which is a nine-item self-report questionnaire referring to the previous two weeks. The answers were coded on a four-point scale ranging from *not at all* (0) to *nearly every day* (3). The PHQ-9 has been shown to have good psychometric properties (e.g. Cronbach’s α = 0.83; Cameron, Crawford, Lawton, & Reid, 2008).

Anxiety symptoms were assessed with the Generalized Anxiety Disorder Assessment (GAD-7; Spitzer, Kroenke, Williams & Löwe, 2006) which is a seven-item self-report questionnaire referring to the previous two weeks. The answers were coded on a four-point scale ranging from *not at all* (0) to *nearly every day* (3). The GAD-7 has been shown to have good psychometric properties (e.g. Cronbach’s α = 0.88; Beard & Björgvinsson, 2014).

The Post-Traumatic Checklist–Civilian Version (PCL-C; Weather & Ford, 1996) is a 17-item self-report measure designed to evaluate symptoms of post-traumatic stress disorder (PTSD) based on criteria outlined in the DSM-IV. Since the DSM-5 was published in 2013 a corresponding PCL-5 has been developed, however, it was agreed that the PCL-C would be used in this study, as this was the outcome measure for PTSD being used at CS at the time. The PCL-C has well documented psychometric properties (e.g. Cronbach’s α = 0.95; Blanchard, Jones-Alexander, Buckley, & Forneris, 1996).

Lastly the AUDIT Alcohol Consumption Questions (AUDIT-C; Bush, Kivlahan, McDonnell, Fihn & Bradley, 1998) is a three-item screening instrument for harmful alcohol consumption. Scores for each question range from 0 to 4, with higher scores indicating greater alcohol use. The AUDIT-C has been shown to have reliable psychometric properties (e.g. Cronbach’s α > 0.79; Ewing, 1984).

#### 1.4.2. Semi-structured interview

A semi-structured interview schedule was designed following a review of a similar qualitative study (Murphy et al., 2014) and discussions within the research team about what adaptations would need to be made to answer the research questions specific to this research study. Seven open-ended questions aimed to understand participants’ experiences of mental health difficulties and the pathways they had taken to accessing services, including which factors enabled them to do so and how they overcame potential barriers. The draft interview schedule was piloted on the first two participants, following which the research team agreed that the interview questions sufficiently elicited data relevant to the research questions and therefore no changes were made and data from the two pilot interviews was included in this study.

### 1.5. Procedure

Recruitment was carried out between January and September 2016. At LVS, staff were introduced to the study and requested to invite patients on their caseload who met the inclusion criteria to participate. After initial consent had been given for their details to be passed on, potential participants were contacted by telephone to discuss the study, to gain an address to post study information (an information sheet, informed consent form and the standardized questionnaires) and to arrange a date and time for the telephone interview. At CS a researcher (H.M.) attended a therapy group at the residential centre to introduce the study directly to potential participants, seek informed consent, complete the standardized questionnaires and arrange a date and time in the following week for the telephone interview. All standardized measures were completed by the participants and given or posted to the researcher and all interviews were conducted over the telephone and audio-recorded.

### 1.6. Analysis

The first stage of the analysis involved collating all of the demographic details and data from the standardized measures (PHQ-9, GAD-7, PCL-C, AUDIT-C). Analysis of the qualitative data occurred in a number of simultaneous stages in accordance with guidelines for conducting grounded theory (Bryant & Charmaz, 2007). The first stage involved the researcher writing memos immediately after the interview, transcribing the interview and listening back to the interview in order to become familiar with the data. The next stage involved openly coding the data by working through transcripts and identifying potential themes using a qualitative analysis program (NVivo, 2014) and simultaneously hand-drawing mind maps. After the first few interviews had been transcribed and analysed, initial codes were merged and reduced using a constant comparative method. As central codes became increasingly clear, all of the transcripts were then re-examined to ensure that no relevant data related to these codes had been missed. Consideration for theoretical sampling, a concept central to grounded theory, was on going throughout data collection and data analysis. At the point at which no new data appeared to be emerging from the interviews the research team met and, following a discussion, it was agreed that theoretical saturation had been reached. Following this, a second researcher (L.R.) analysed two randomly selected transcripts to ensure inter-coder reliability. A discussion between H.M and L.R highlighted agreement on all of the major codes and on the idea that there were two discrete stages to help-seeking suggesting a high level of inter-rater agreement. Differences between the researchers were related to linguistics rather than semantics and these were resolved through discussions. Lastly the research team met to agree on the final codes and all of the transcripts were re-examined again to ensure no relevant data had been missed.

## 2. Results

### 2.1. Sample characteristics

Eighteen veterans from LVS (*n =* 8) and CS (*n = *10) agreed to take part in the study. No one dropped out of the study, nor did anyone request to withdraw their data. However, one participant’s data was excluded from the study in order to have a more homogenous sample, as they were the only female and participant of officer rank. Therefore, the final sample consisted of 17 male veterans for whom their demographic information is displayed in Table 1.

**Table 1.** Socio-demographic characteristics of the sample.

Participant	Age	Ethnicity	Employment status	Relationship status	Children	Rank at exit	Service	Years in Armed Forces	Years since retiring
1	56	White British	Signed off sick	Single	Yes	Corporal	Army	18	22
2	32	White British	Signed off sick	In a relationship	Yes	Senior Aircraftsman	RAF	9	5
3	43	White British	Retired	In a relationship	No	Sergeant	Army	15	12
4	56	White British	Retired	In a relationship	Yes	Sergeant	Army	13	12
5	48	White British	Signed off sick	Married	Yes	Corporal	Army	14	6
6	53	White British	Self-employed	In a relationship	No	Guards Man	Army	8	29
7	33	White British	Full time	Married	Yes	Private	Army	13.5	3
8	57	White British	Signed off sick	Single	No	Corporal	RAF	22	17
9	47	White British	Unemployed	In a relationship	No	Private	Army	6	24
10	57	White British	Full time	Married	Yes	Lance Corporal	Army	13	25
11	68	White British	Retired	Single	Yes	Corporal	Army	8	40
12	68	White British	Self-employed	Single	Yes	Private	Army	5	47
13	39	White British	Unemployed	Single	Yes	Corporal	Army	7	11
14	43	White British	Full time	Married	Yes	Lance Corporal	Army	6	21
15	57	White British	Unemployed	In a relationship	Yes	Sergeant	Army	15	27
16	53	White British	Unemployed	Single	Yes	Private	Army	5	31
17	60	Black Caribbean	Unemployed	Single	No	Corporal	Army	9	36

All participants were from non-commissioned ranks and the majority were white British (16/17), not currently working (12/17), had children (12/17) and had been part of the Army (15/17). The mean age of the sample was 51 with a large range of 32–68. Length of service varied from 5–22 years, with the mean length of service being 11 years. The time since leaving the AF varied from 3–47 years. At the time of interview participants had either just completed treatment (within the last four weeks) or were currently receiving treatment for mental health difficulties.

### 2.2. Results of standardized measures

Rates of mental health disorders are reported in Table 2. Eighty-eight percent of participants reported clinically significant levels of depression (score ≥ 10) and 71% of participants reported clinically significant levels of anxiety (score ≥ 10) according to the PHQ-9 and GAD-7, respectively. In addition, 47% of participants met criteria for PTSD according to the PCL-C (score ≥ 50) and 59% of participants scored above threshold for alcohol difficulties on the AUDIT-C (score ≥ 5). There was a large variance in scores between participants for alcohol use, which may be related to the no-alcohol policy that CS has during an individual’s residential stay. According to independent measures *t*-tests, there were no significant differences in scores on the standardized measures between veterans attending CS compared to veterans attending LVS.

**Table 2.** Scores from standardized measures.

Participant	PHQ-9	GAD-7	PCL-C	AUDIT-C
1	15*	16*	70*	4
2	26*	21*	67*	5*
3	25*	21*	80*	7*
4	19*	14*	71*	12*
5	17*	15*	62*	1
6	15*	18*	46	0
7	9	10*	34	2
8	20*	21*	70*	9*
9	10*	7	20	6*
10	10*	7	37	0
11	10*	5	33	6*
12	9	7	42	7*
13	13*	16*	66*	5*
14	12*	11*	48	7*
15	17*	6	42	12*
16	23*	20*	73*	4
17	20*	10*	48	0

* = clinically significant level of depression and anxiety or above threshold for PTSD or alcohol problems

### 2.3. Results of qualitative analysis

Analysis of the data highlighted two stages to help-seeking for veterans, with specific barriers and enablers to help-seeking at each stage; these are displayed in Table 3. ‘Initial help-seeking’ refers to the stage at which an individual recognizes that there is a problem and makes the first move towards seeking help. ‘Pathways through treatment’ is the stage at which an individual attempts to actively engage with a professional service.

**Table 3.** Barriers and enablers to initial help-seeking and pathways through treatment.

Domain	Theme	Number of participants	Supporting quotations
Barriers to help-seeking	a. Insight/readiness to change	17	‘I wasn’t sure that there was something wrong’ [P14] ‘I thought I had irritable bowel syndrome, high blood pressure and stress … but as its transpired over the years … I had Post-Traumatic Stress Disorder’ [P10] ‘I realized there was a problem there, but I didn’t know who to turn to’ [P11]
	b. Self-stigma	13	‘I felt very embarrassed’ [P8] ‘I just thought I was going mad’ [P15] ‘It was a weakness’ [P13] ‘I thought I was a loser … you know I was useless’ [P2]
	c. Public stigma	15	‘You say PTSD and people think you’re a walking time bomb’ [P13] ‘Civilians do not have a clue, they don’t understand and straight away they will judge’ [P3] ‘It worried me that everybody would think that I was bluffing it, even though I wasn’t, so I just carried on and on and on’ [P5]
Enablers to help-seeking	d. Internal resources	14	‘I’m thinking I’m going to give myself a bit of a better chance’ [P6] ‘Laughter does help you get through these kind of things’ [P14] ‘My willpower at the end of the day’ [P10]
	e. Media/advertising	9	‘I literally just kind of googled it’ [P1] ‘I’m still on servicemen networks through Facebook’ [P8] ‘You’ll be amazed what you can learn off the TV’ [P3]
	f. Risk of not help-seeking	17	‘I was putting myself in some very dangerous situations, with some highly dangerous people, and I didn’t care about it’ [P4] ‘I said [to myself] “if you don’t get help here, Mate, you’re going to prison”’ [P8] ‘I tried to take my life’ [P1] ‘I lost my job, um, I lost my flat, and in fact, I was homeless’ [P17] ‘I was trying to commit suicide at least 3–4 times per week’ [P10]
	g. Support from others	14	‘I had a very supportive wife all the way through’ [P10] ‘I’ve had a lot of encouragement off friends, my partner’ [P3] ‘A couple of my best mates that I grew up with just dragged me down there one day and said “look you’ve got to sort this man out”. Interviewer: And would you say that without that support network you probably wouldn’t have got help when you did? Participant: I’d be dead by now, I know that. There’s no ifs and buts about that’ [P6]
Barriers to pathways through treatment	h. Practical	11	‘It took three months to see a nurse, three months to see a psychiatrist by which point things are getting worse’ [P1] ‘When you ring sometimes they don’t ring you back’ [P7] ‘Initially it was a phone call saying what’s the matter … it was very cold, there was absolutely no empathy’ [P4] ‘I sometimes have issues on public transport … I was driving, but I just found I was getting wound up on the way there, so I thought the only other way to get there is the tube which I don’t use during busy hours’ [P13]
	i. Health services and professionals	17	‘He just dismissed those [flashbacks] as night terrors’ [P1] ‘It’s not that they don’t want to help you, it’s that they don’t understand what to do’ [P10] ‘I wasn’t being treated for my problems’ [P9] ‘Uncaring temporary staff, and an absolute lack of any kind of therapy … no occupational therapy, no group therapy, no one-to-one, nothing at all’ [P1]
Enablers to pathways through treatment	j. Diagnosis	15	‘A welcome relief for me, it was absolutely inspiring for me, it changed my life’ [P15] ‘It was like a balloon being let down, all that pressure gone, the weight was lifted’ [P9] ‘It just clarifies things’ [P16] ‘It gave me justification that I wasn’t going completely insane’ [P1]
	k. Health services and professionals	17	‘I went to see my GP, um, who was amazing’ [P1] ‘He looked on the internet at the time’ [P7] ‘They gave me a number to ring up all the time, I kept getting the weekly visits from the regional welfare officer, and probably every couple of months from the community practice nurse … Nothing was too much trouble for them. Absolutely nothing’ [P10] ‘She’s really understanding … they were so quick and straight in’ [P15]
	l. Military environment	17	‘I could take it from him being an ex-major in the Army, and it was the first time I had heard an Army guy say to me “it’s nothing to be ashamed of, it’s not your fault, let X sort it out for you”, and that was sort of easier for me to take on board’ [P11] ‘She seemed to understand a lot of military terms, so she got it where I was coming from’ [P12] ‘It was very eye opening, all of a sudden there were a whole bunch of guys that were just like me, that were human beings that had thought they were indestructible, um, and that for a whole gamut of different reasons, had kind of fallen down … And the staff there were incredible … I realized that I kind of wasn’t alone, and that there was a whole world out there of people that were struggling with similar issues’ [P1]

*Stage 1: Initial help-seeking: Barriers*

1. Insight/readiness to change

All participants described challenges in recognizing that they were suffering from mental health difficulties, despite experiencing severe symptoms. A number of participants believed that nothing was wrong whereas other participants described ignoring or minimizing their symptoms or not knowing where to seek help. Three participants described misattributing their symptoms to physical health-related difficulties rather than mental health difficulties.

P10: I thought I had irritable bowel syndrome, high blood pressure and stress … but as it’s transpired over the years … I had post-traumatic stress disorder.

- Self-stigma

Thirteen participants described negative internal beliefs such as ‘shame’ and ‘embarrassment’ which acted as a barrier to help-seeking. Eight participants also talked about feeling ‘weak’ and ‘inadequate’.

P2: I thought I was a loser.

- Public stigma

Most participants had anticipated negative judgments from others which initially prevented them from seeking help.

P3: Civilians do not have a clue, they don’t understand and straight away they will judge.

Eight participants understood these fears as resulting from negative experiences of help-seeking during their time in the AF. Other participants feared being accused of ‘malingering’ and one participant feared being arrested which prevented them from seeking help.

P5: It worried me that everybody would think that I was bluffing it, even though I wasn’t, so I just carried on and on.

*Stage 1: Initial help-seeking: Enablers*

Despite the aforementioned barriers, participants in this study had engaged with treatment, and they also identified various enablers that helped them to seek help.

- Internal motivation

Nine participants identified internal resources such as motivation to get well and regain control over their lives, hopes for the future and using humour to overcome self-stigma as enabling factors to help-seeking.

P6: I’m thinking I’m going to give myself a bit of a better chance.

- Media/advertising

Nine participants found internet searches, leaflets, television programmes and magazine stories helped them to identify what they were suffering from, where to go to seek help and to realize that they were not alone in experiencing symptoms, which in turn enabled help-seeking.

P3: You’ll be amazed what you can learn off the TV.

- The risk of not help-seeking

All participants described seeking help at a time of crisis. In this study, crisis points generally occurred if the veteran was suicidal, at risk of going to prison or at risk of losing their family or job.

P10: I was trying to commit suicide at least three-to-four times per week.

- Support from others

Support from partners, military organizations, friends, family or a fellow veteran was another enabling factor to help-seeking for 13 participants.

Interviewer: And would you say that without that support network you probably wouldn’t have got help when you did?

P6: I’d be dead by now, I know that, there’s no ifs and buts about that.

*Stage 2: Pathways through treatment: Barriers*

- Practical barriers

Practical barriers that delayed help-seeking emerged in 11 of the transcripts. These included lengthy waiting times, especially when being referred between services, limited session numbers and the lack of veteran-specific services within the NHS. In addition, three participants recalled negative experiences of a telephone-based mental health assessment, which led them to disengage from the service.

P4: Initially it was a phone call saying what’s the matter … it was very cold, there was absolutely no empathy or understanding there you know, and I thought wow, this is going to be tough.

Four participants described barriers specific to attending inpatient treatment which included fears about being among other veterans which may trigger flashbacks and having to be away from family. In contrast, seven participants felt that being among other veterans in an inpatient setting enabled them to feel safe because of their shared past experiences. Two participants described barriers specific to outpatient treatment which included having to take public transport or drive to attend appointments and not being able to get time off work.

P13: I sometimes have issues on public transport. I was driving, but I just found I was getting wound up on the way there, so I thought the only other way to get there is the tube which I don’t use during busy hours.

- Poor therapeutic relationship

The majority of participants described negative past experiences related to accessing mainstream NHS services. Among all of the participants there was a perception that health professionals within mainstream NHS services lacked necessary military-specific knowledge and terminology to help veterans. Four participants described difficult therapeutic relationships with health professionals, which left them feeling invalidated and caused them to disengage from treatment. The content of the therapy also effected a number of participant’s engagement. For example, two participants described fears of traumatizing the therapist with their ‘war stories’, three participants described a fear of being unable to cope with talking about their traumatic experiences, and two participants were disappointed not to have the opportunity to talk about their traumatic experiences.

P10: It’s not that they don’t want to help you, it’s that they don’t understand what to do.

*Stage 2: Pathways through treatment: Enablers*

- Receipt of a diagnosis

The majority of participants identified receiving a diagnosis as a significant enabler in their pathway to help-seeking due to the ‘relief’, ‘clarity’ and ‘understanding’ it provided. Three participants felt that receiving a diagnosis of PTSD meant that they were not going ‘insane’.

P15: It was a welcome relief for me, it was absolutely inspiring for me, it changed my life.

In contrast two participants described feeling stigmatized by an initial diagnosis of depression or adjustment disorder but feeling relieved when the diagnosis was updated to PTSD.

P1: The psychiatrist there, um, challenged the PTSD thought … he just dismissed those [flashbacks] as night terrors.

- Flexible health services and professionals

Participants identified a number of factors specific to health services and professionals that eventually made it possible for them to seek help. These included on-going support from a GP or a support worker/welfare officer for seven participants, being able to access services or transfer between services quickly, and service flexibility around cancellations, missed appointments and session numbers.

P10: They [CS] gave me a number to ring up all the time, I kept getting the weekly visits from the regional welfare officer, and probably every couple of months from the community practice nurse … Nothing was too much trouble for them.

- Military therapeutic environment

All participants valued being treated for their mental health difficulties within an environment specifically for veterans, by a perceived specialist health professional. Recurring themes included professionals’ use of military terminology and the military style banter between veterans, which allowed participants to feel safe, normal and to trust others.

P1: I realized that I kind of wasn’t alone, and that there was a whole world out there of people that were struggling with similar issues.

#### 2.3.1. What might be done to improve veterans’ help-seeking and pathways to care?

Numerous suggestions emerged regarding how to improve the help-seeking experiences of veterans in the future. Participants identified the following four areas where improvements could be made: (1) military resettlement procedures, (2) educating health professionals about the military context, (3) increasing public awareness about veterans’ mental health and available specialist services through advertising and (4) employing and training veterans to support other veterans with mental health difficulties to seek professional help.

## 3. Discussion

### 3.1. Summary of findings

This study aimed to investigate the barriers and enablers to help-seeking from the perspectives of veterans who have successfully accessed treatment, and to understand their views on what might be done to increase veterans’ help-seeking for mental health difficulties in the future. This study identified two stages to help-seeking – initial help-seeking and pathways through treatment – with different barriers and enablers at each stage. Barriers to help-seeking included recognizing that there is a problem, stigma-related beliefs, negative attitudes towards health care and practical barriers. Enablers to help-seeking included being in crisis, social support, motivation, the role of the media, receiving a diagnosis of PTSD and veteran-specific services.

A common theme for all participants, found frequently in previous research, was reaching a crisis point prior to seeking help (Zinzow, Britt, Pury, Raymond & Burnette, 2013). One reason for reaching a crisis point seemed to be veterans’ inability to recognize their own mental health difficulties. Hence, research suggests that gaining psychological understanding about one’s mental illness can enable help-seeking (Murphy et al., 2014). The current study found that one way of improving veteran’s knowledge about mental health difficulties is through films, advertisements and the internet. Web-based public health interventions are beginning to be developed in order to address key barriers to mental health care for veterans (Kuhn, Drescher, & Hoffman, 2013) with some evidence of their efficacy (Whealin, Kuhn, & Pietrzak, 2014). In addition, in the UK and the US a number of mobile applications (apps) have been designed for military personnel and veterans such as ‘Veterans Mental Health’ (South Staffordshire and Shropshire Healthcare NHS Foundation Trust, 2015), which aim to provide information on mental health problems and highlight where to get help.

Study participants who were able to recognize that they were unwell described a sense of motivation to seek help in order to regain control over their lives. Contrary to these findings, some research with veteran populations has found that stoicism-related beliefs and behaviours can prevent help-seeking (Zinzow, Britt, McFadden, Burnette, & Gillispie, 2012). Hence, future help-seeking interventions may benefit from highlighting the risks of coping with one’s mental health difficulties alone and wish to portray seeking help as an act of strength and a positive movement towards problem solving. Other participants who were able to recognize that they were unwell described how stigma-related beliefs and negative attitudes towards the outcome of seeking help were a barrier to help-seeking, as has been well evidenced in previous research (Sharpe et al., 2015).

Support from a significant other was found in this study to have a positive influence on veterans’ decision to seek help, as is well documented in the research literature (e.g. Warner et al., 2008). However, similar to previous findings, practical barriers such as long waiting times and having a telephone assessment acted as a further barrier to accessing help (e.g. Damron-Rodriguez et al., 2004). Participants in this study reported more practical issues when accessing mainstream NHS mental health services compared to specialist veteran services. Attending a specialist service was a highly endorsed enabling factor to help-seeking for all participants, as has been found in previous research with veterans (NHS England, 2016). One element of this was that veterans valued the military specific knowledge related to military service and mental health that health professionals had in this environment. The importance of the therapeutic relationship in treatment engagement has been well documented in previous research with veterans (Creamer & Forbes, 2004) and was further endorsed by participants in this study. A recent systematic review by Kantor, Knefel, and Lueger-Schuster (2017) cited a barrier to help-seeking specific to trauma survivors of being concerned about re-experiencing the traumatic events, as was found in this study, further endorsing the importance of a good therapeutic relationship.

The majority of participants found receiving a diagnosis of PTSD helped them to seek treatment. Evidence suggests that having a diagnosis provides reassurance that an individual’s situation is not unique and hence reduces inappropriate feelings of blame, and helps individuals to communicate their difficulties to others whilst also allowing them actively to seek the most relevant treatment (Craddock & Mynors-Wallis, 2014). However, in this study some participants held negative beliefs about being given a diagnosis of depression or adjustment disorder. Previous research with military personnel has found that if individuals believe that they are accountable for their symptoms, or that society may hold them accountable for their symptoms, then they are likely to experience a stronger sense of stigma and as a consequence to forgo help-seeking (Britt, Greene, Castro, & Hoge, 2007). Future research should focus on exploring the role of diagnosis in help-seeking for mental health difficulties in veterans and consider how any diagnosis-related barriers to help-seeking may be overcome.

Lastly veterans identified a number of factors as important when considering future service provision. Recommendations included making improvements to military resettlement procedures; educating health professionals about veterans’ healthcare needs; increasing public awareness of veterans’ mental health and advertising available services; and employing and training veterans to support other veterans with mental health difficulties. These goals map to the work being done by a number of current initiatives such as specialist leaflets informing GPs about veterans mental health needs have been disseminated (‘Meeting the healthcare needs of a veteran’, 2011); a proposal for an increase in NHS specialist services for veterans is underway (NHS England, 2016); and a current national mental health campaign called ‘Heads Together’ has a particular focus on veterans among other specific groups. In future such interventions and health services may benefit from employing and training veterans as a powerful way of targeting veteran’s negative beliefs about mental illness and treatment (Dickstein, Vogt, Handa, & Litz, 2010).

### 3.2. Limitations

There are a number of limitations to this study. This study was designed using grounded theory and hence the sample was small and therefore it was not intended that findings would be generalized to all help-seeking veterans suffering from mental health difficulties. Despite this the sample included was all male, from non-commissioned ranks and had served in either the army or the RAF. Therefore, future research would benefit from exploring barriers and enablers to help-seeking for other veteran groups such as servicewomen and individuals from officer ranks. A further limitation of this study was that the majority of participants had previously accessed mainstream NHS services where they reported negative experiences, prior to accessing help from specialist veteran services, which may have biased their responses. It is likely that a significant number of veterans successfully access help for mental health difficulties from mainstream NHS services but their experiences were missed in this study because participants were recruited from veteran-specific services only. In addition, symptoms of PTSD were measured using the PCL-C for convenience, however, this corresponds to the DSM-IV diagnostic criteria for PTSD and not the DSM-5 and therefore it should be acknowledged that it is an out-dated measure and that in future research it would be more relevant to use the PCL-5. Future research should explore the barriers and enablers to help-seeking for veterans accessing help from mainstream NHS services. Lastly all of the interviews were conducted over the telephone and while participants were generally positive in their feedback about their experience of the interview, evidence suggests that participants can feel nervous when talking over the phone, which may impact on their ability to openly share very personal experiences (Fear, Van Staden, Iversen, Hall, & Wessely, 2010).

### 3.3. Conclusions

This study provided an in-depth insight into important barriers and enablers to help-seeking at different stages in the care pathway for veterans suffering from mental health difficulties. Previous research has focussed on barriers to help-seeking and similar to past findings this study found that factors such as stigma, attitudes towards healthcare, not recognizing mental health difficulties and practical issues were all barriers to help-seeking for veterans. Less cited in previous research was the finding that a fear of being accused of malingering was a key barrier to help-seeking. Enablers to help-seeking found in this study and previous research included support networks, identifying mental health difficulties, and severity of symptoms. In addition, this study found that the media, diagnosis, motivation and accessing help from veteran-specific services were all important enablers to help-seeking for this group. Participants made valuable suggestions regarding how we may improve help-seeking in veterans in future and it is promising to see a number of initiatives utilizing technology currently underway.
